# Hepatocellular Carcinoma: Focus on Different Aspects of Management

**DOI:** 10.5402/2012/421673

**Published:** 2012-05-13

**Authors:** Sene Waly Raphael, Zhang Yangde, Chen YuXiang

**Affiliations:** National Hepatobiliary and Enteric Surgery Research Center of Ministry of Health, Central South University, Changsha, Hunan 410008, China

## Abstract

Hepatocellular carcinoma (HCC) is the fifth most common cancer and the third cause of cancer-related mortality worldwide. Its incidence is clearly arising comprised by the prevalence of major risk factors mainly hepatitis B and hepatitis C. The population at risk is composed of chronic liver patients at the stage of extensive fibrosis or cirrhosis. The monitoring programs of this population have allowed early detection of disease management to promote a radical therapy. Understanding the carcinogenic process and the mastery of the staging systems remain essential keys in diagnosis and treatment of HCC. Recent advances in diagnosis and new treatments have made important impacts on the disease by increasing survival rates and improving quality of life for HCC patients. This paper outlines the different management aspects of HCC which include epidemiology, prevention, carcinogenesis, staging systems, diagnosis, surveillance, and the treatment.

## 1. HCC Epidemiology

**Figure 1:**
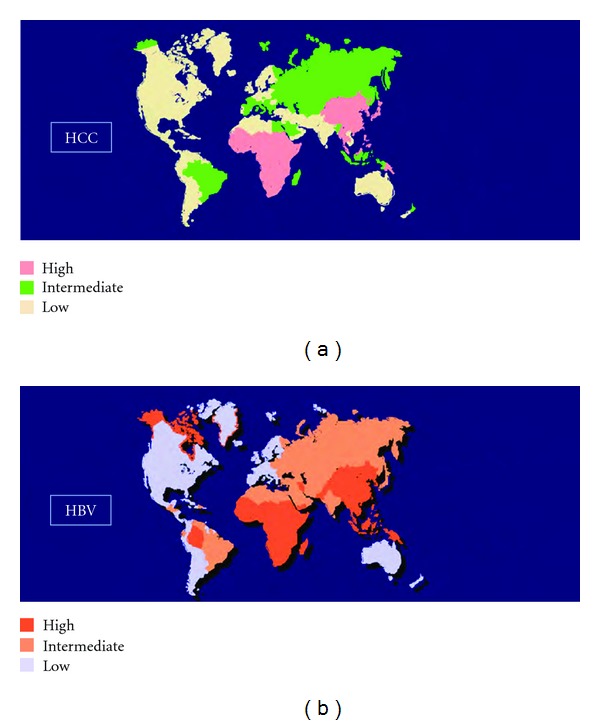
The striking parallel geographical distribution of the incidence of chronic hepatitis B virus infection and that of hepatocellular carcinoma. (Source [6] with permission of the Pathologie Biologie Journal.)

HCC is the most common primary liver cancer. The annual number of new cases of HCC worldwide is over one million, making it the 5th most common cancer worldwide and the 3rd leading cause of cancer-related death, preceded only by the lung and stomach cancers [1–6]. The global distribution varies by region due to factors at the origin of the disease. HCC is an end result of some chronic infections with the hepatitis B (HBV) or the hepatitis C (HCV) Figure 1 [7–9]. More than 80% of HCCs develop in Asian and African countries where between 40% and 90% of HCCs are attributable to chronic hepatitis B [1, 10]. China especially comprises more than half the rate of new cases recorded with over 55% (around 120 million people in China are carriers of the HBV corresponding to almost a third of people infected worldwide [11, 12]. In Singapore, Japan, and Australia/New Zealand, HCC infection is exceptionally due to the high incidence of HVC infections [13–15]. Prevalence of HCV infections is reported to be the main leading cause of HCC in Europe and also in the United States where the incidence is relatively low. Currently, there are an estimated 3 million people in United States with chronic hepatitis C; these patients are estimated to develop HCC at a rate of 0.5% to 5% per year [12]. In Europe, the incidence of HCV may be related to the extensive campaign to vaccinate children in the years 1940s to 1950s and possibility to inadequate sterilization of nondisposable needles and syringes up to the mid-1970s. Those infected with hepatitis C during this period have now been infected for 30 years and therefore have significantly increased risk of having established cirrhosis. Thus, the silent hepatitis C epidemic from that area is the likely cause of the increase in HCC incidence in those parts of the world [16]. In Africa, exceptionally in Egypt, HCV is the most leading increase in HCC. Egypt is known for being the country in the world where the rate of HCV is higher, about 24% of the people are estimated to carry HCV and the more than 50% of blood donors have anti-HCV in some towns [17]. Between 1993 and 2002, there was an almost twofold increase in HCC amongst chronic liver patients [2]. Men are more likely to be affected than women with HCC [18]. This trend was observed in almost all countries. Therefore, it was noted in some European countries such as Switzerland (male : female: 4 : 1), Italy (male : female: 5 : 1), France (male : female: 5 : 1); in the developing states, the ratio is more equal, for example, China 3 : 1, Gambia 2.8 : 1, and Zimbabwe 2.4 : 1 [5, 19]. In Japan, the male/female ratio was 4.5 between 1984 and 1985 and 2.5 between 2002 and 2003 [15]. The reasons of these trends are not well understood but several factors may explain that. Males are more likely to be infected with HBV and HCV, in addition cigarettes smoker, and alcohol consumer have a higher body mass index (BMI). Testosterone rate has been shown to correlate with HCC indicating a probable role for the sex hormones in the development of HCC. IL-6 is thought to be implicated, as IL-6 disruption abolished the gender differences in hepatocarcinogenesis [3, 5, 19].

### 1.1. Risk Factors

Besides HCV and HBV, other risk factors have been reported to be involved in the development of HCC. Any agent leading to chronic injury and eventually cirrhosis constitutes an oncogenic agent [20]. Aflatoxin (AF), alcoholism, and nonalcoholic steatohepatitis (NASH) are important and prevalent in certain areas of the world.

### 1.2. Cirrhosis

Cirrhosis is an end stage of chronic diffuse liver disease. It is characterized by alteration of the normal liver into structurally abnormal nodules of liver cells surrounded by fibrosis. The changes must be diffuse throughout the liver. The risk of cancer development from chronic or cirrhosis varies according to the degree of fibrosis [21]. Main causes of liver cirrhosis are alcohol use, chronic hepatitis B and C, and nonalcoholic steatohepatitis [22–25].

Cirrhosis is present in 80–90% of HCC patients and is thereby the largest single risk factor [19]. With exception of HBV and AF, all other etiological risk factors are associated with cirrhosis. It is generally believed that the majority of HCCs develop in a progressively from acute hepatitis through various stages of chronic hepatitis, to cirrhosis, to HCC [26].

### 1.3. Alcohol Intake

The frequency of HCC in alcoholics is rather low and far less than that of tumors of the upper digestive tract [27]. No agreement exists on the dose-effect relationship between alcohol intake and risk of HCC, but some experts defined it as a proven risk factor for HCC when the daily consumption is estimated at 50 to 75 g per day. It has not been proven effective carcinogen direct alcohol on the occurrence of HCC, but rather an indirect effect through repeated injuries progressing to extensive fibrosis and cirrhosis. Some mechanisms such as chromosomal loss, oxidative stress, a decreased retinoic acid level in the liver, altered DNA methylation, and genetic susceptibility can be regarded as causes of alcohol leading to HCC [28–39]. Alcohol, because of its increasing intake in many countries, may continue to be an obvious cause of HCC development in the world.

### 1.4. Aflatoxin (AF)

Aflatoxin is believed to be a major causative agent in the high incidence of primary liver cancer seen in certain regions of the world. In some Africa and Asia regions especially, it is known to be a key risk factors of HCC. Aflatoxins are a group of approximately 20 related fungal metabolites with four major known as B1, B2, G1, and G2. Among them, B1 is the most potent naturally occurring chemical liver disease carcinogen known [31]. They are produced by *Aspergillus flavus* and related fungus that grow on improperly stored foods, such as corn, rice, and peanuts. Effects of aflatoxin carcinogen result in p53 gene mutations. Generally, in human cancer, in more than 50% of tumors, p53 is mutated and these mutations occur at the third position of codon 249 with the GC—TA transversion [32, 33]. Associated with HBV, dietary exposure to aflatoxin increase the HCC risk factors [34].

### 1.5. Nonalcoholic Steatohepatitis (NASH)

Nonalcoholic steatohepatitis was originally believed to be a benign disease, but it has been recently revealed that NASH could lead to irreversible liver diseases [35]. NASH is a term used to describe a spectrum of conditions characterized by histological findings of hepatic macrovascular steatosis with inflammation in individuals who consume little or no alcohol [36]. It is part of the spectrum of nonalcoholic fatty liver disease (NAFLD) that can lead to cirrhosis in patients without a history of alcohol abuse and whose prevalence is increasing in Western countries because of the obesity and diabetes mellitus incidence. 20% of NASH cases generally progress to cirrhosis and result in complications including HCC [37, 38]. Hedgehog (Hh) pathway activation and NKT (natural killer T) cells seem to be involved in the development of NASH-related liver fibrosis. The liver's response to damage due to fat accumulation is modulated by these cells. Bugianesis [39] proves that the activation of the liver NKT cells generates soluble factors that promote fibrogenesis via a mechanism involving myofibroblastic activation of hepatic stellate cells.

## 2. HCC Prevention

 Since chronic viral B and C are known to be the most common causes of carcinogenesis, prevention of HCC requires an effective risk control of infection due to these factors. Therefore, vaccination against the hepatitis B virus appears the best way for prevention [40–42]. In chronic HBV infection, mother-to-child transmission generally accounts for 35 to 50% through exposure to blood or blood-contaminated fluids at or around birth. Approximately 70 to 90% of the infants of the HBeAg-seropositive carriers mothers became HBsAg carriers [13, 43]. Routine vaccination at birth in countries with high prevalence of HBV infection can reduce the transmission. In Taiwan, where the world's first universal hepatitis vaccination program was conducted in July 1984, chronic HBV infection in mother-to-infant transmission has decreased from 86–96% to 12–14% [44]. As a consequence a reduction in the incidence of HCC was noted. Success in mass program vaccination has been seen in Thailand and Singapore [45]. In western countries, universal vaccination has been adopted, and, in developing countries, mass vaccination programs are being introduced. These programs should lead in coming decades to significant decrease in HCC incidence [3]. In case of persons already infected chronically with a HCC risk development, appropriate way is to select patient at risk for treatment and not a mass vaccination [46].

There is no vaccine available for HCV due to its high mutation during viral replication. Prevention of HCV infection requires the reduction of risky behavior and the improvement of hygiene by the widespread use of disposable syringes and needless reduction of transmission among injecting drug users and also the screening of blood donors and organ donors [47, 48].

Reduction of alcohol consumption in HCV-infected patients, controlling obesity and diabetes mellitus, limiting fungal contamination of crops either pre- or postharvested to reduce aflatoxin exposure are also measures that could have a real effect on HCC risk.

Some chemoprevention agents are also used in the HCC prevention. They differ by country but also the underlying disease and the results are unreliable according to the desired effect. They are used to prevent cirrhosis when HCC in non-HCV-related cirrhosis prevention has been unachievable [3]. Interferon therapy is one of the most used of these agents against HCC development but their effects are controversial; studies performed in USA showed that interferon therapy seems to fail to reduce HCC incidence while in Japan studies demonstrated a HCC incidence reduction in interferon-treated patients [40]. In China, the combination of lavamidin and ravadin demonstrated promising results in HCC prevention although the study focused on a reduced patient sample. Many other agents are in trials, and results should help to better prevent HCC in certain population groups at risk.

## 3. Molecular Mechanisms of Liver Carcinogenesis

 In humans, 90% HCC cases arise as complication of chronic liver disease/cirrhosis with fibrosis playing a major predisposing role [42]. Molecular mechanisms that lead to the development of HCC are not well known, but much research is being conducted to better understand these processes. So the development of molecular biology has enabled significant progress to improve the knowledge on molecular mechanisms of carcinogenic. HCC is known to be a result of the evolution process of a large number of genetic and epigenetic alterations that some are observed at neoplastic stages [20, 28, 42, 49]. These alterations affect the proteins in certain major signaling pathways that control the cycle, proliferation, and cell survival.

### 3.1. Wnt-*β*-Catenin Signaling Pathway

Mutations in this pathway have been described in 20 to 40% of HCC. Wnt-*β*-catenin signaling pathway plays a role in all phases of liver development and maturation which are stem cell renewal, zonation, cell adhesion, proliferation, differentiation, liver regeneration, and epithelial-mesenchymal transition [50–52]. HCC occurs frequently through mutations in the N-terminal region of *β*-catenin that stabilizes the protein and permits an elevation of constitutive transcriptional activation by *β*-catenin/TCF complexes [53]. *β*-catenin mutations seem to be correlated to the etiology of the HCC. In HBV- and HCV-related HCC *β*-catenin mutations, rates are still subject to discussion. In patients with HBV, *β*-catenin mutations are found in lower frequency, unlike HCV-related HCC where the rate is high at over 40%. In patients without HBV, the mutations are associated with chromosome stability and genetic alteration [50, 54].

### 3.2. Activation of the Insulin-Like Growth Factor (IGF) Signaling Pathway

It is also involved in occurrence of HCC. The IGF system consists of two ligands, IGF-I and IGF-II; three cell-membrane receptors, IGF-I receptor (IGF-IR), insulin receptor (IR), and IGF-II receptor (IGF-IIR); six high-affinity IGF binding proteins, IGFBP-1 through 6 [55]. An overexpression of the IGF-1 and IGF-2 receptor (IGF-1R/2R) and silencing of the IGF-binding proteins (IGFBP-1-5) lead to cascade of molecular events such as cell proliferation, antiapoptosis and invasive behavior [56]. The activation of this signaling pathway in HCC is initiated by the IGF II by a loss of promoter-specific imprinting and reactivation of fetal promoters, reduced expression of IGF-binding protein, and/or activation of the IGF II-2R, which mediates IGF-II degradation. This signaling pathway seems to have therapeutic interest; by blocking the IGF-II overexpression, HCC development can be disturbed and also inhibition of IGF-IR by an antibody or tyrosine kinase inhibitors HCC cell proliferation can be reduced with or without apoptosis increase [57]. 

### 3.3. The P13/PTEN/AKT

 Pathway is involved in several cellular processes such as proliferation, apoptosis, differentiation, cell motility, cell cycle progression, tumor growth, and angiogenesis [56, 58]. In experiment in vitro, this pathway plays a role in HCC cell invasion by enhancing MMP-9 expression [58]. It has also a therapeutic interest by the kinase components of the P13K pathway which are essentially exciting targets for the rational design of small molecules [59].

### 3.4. TP53 Tumor Suppressor Gene

The TP53 mutations are strongly associated to the HCC and considered as the most consistently mutated tumor suppressor gene in HCC. It occurs in 30 to 50% hepatocellular carcinomas [60–62]. In HCC, the TP53 mutations type and frequency are known to be changing according to the geographic regions of tumors. Dietary exposure to aflatoxin B1 (AFB1) (Africa, Asia) induces mutation in codon 249 by G-T transversion and therefore leads to the amino acid substitution R249S [63, 64]. Chronic infection with HBV and HCV viruses, and oxyradical disorders including hemochromatosis, also generate reactive oxygen/nitrogen species that can both damage DNA and mutate cancer-related genes such as TP53 [63, 65]. In geographic locations without AFB1 exposure as Western countries TP53 mutations are found in around 20% of HCCs [50]. 

Except TP53, research is conducted on other genes such as AXIN1 and CTNNB1 to determine the role of their mutations in the occurrence of HCC. Their role seems to be less in HCC because they are found to rarely mutate and then occur in less than 10% of HCC [28, 50, 56].

## 4. HCC Staging

Staging system of cancers for classification of HCC provides a guide to patient assessment and to direct therapeutic interventions [66, 67]. It is then a key to predict the prognosis of patients with cancer, to stratify the patients according to prognostic variables in the setting of clinical trials, to allow information exchange among researchers [68]. However, there is no general consensus in the different system and geographic variations exists [41]. For HCC, several staging systems (Table 1) have been developed and generally take into account tumor characteristics such as tumor size, number, vascular invasion and metastasis, and also the severity of the underlying cirrhosis [1, 69]. For a classification system to allow a reliable assessment of prognosis, two conditions are necessary: prognosis between two stages should be as different as possible (discriminatory ability) and as identical as possible within the same stage (homogeneity). Moreover, the survival of patients in favorable stages must be greater than unfavorable stages (monotonicity of gradients) [70]. Among the staging systems, the most used for the HCC are Okuda classification, TNM classification, CLIP classification, BCLC classification, French classification, CUPI classification, and JIS classification (Table 1).

### 4.1. Okuda Classification

Established in 1985 from 850 cases of hepatocellular carcinoma, Okuda classification is one of the most commonly used staging systems in the world. It includes both parameters related to the tumors stage (more or less than 50% of liver area involved) and functional status such as albumin, ascites, and bilirubin. However, it does not include important prognostic parameters such as the unifocal, multifocal, or diffuse state of the tumor, existence of a portal vein thrombosis or distant metastasis and the alpha-fetoprotein (AFP) rate. It is generally used in patient stratification with advanced or symptomatic stage HCC (Okuda III); so it is unsuitable for the distinction of patients at more favorable prognosis [66, 68, 70]. 

### 4.2. TNM Classification

Proposed as the best staging system to assess outcome of HCC patients undergoing resection and based on tumor size (T1 to T4), number, vascular invasion, regional node status, and distant metastases, TNM is only based on tumor characteristics. It ignores the severity of underlying chronic liver disease, showing poor prognostic in patient undergoing curative treatment. TNM has been revised and has become American Joint Committee on Cancer (AJCC) TNM (Tumor, Node, Metastasis) staging system. Then, the fibrosis is taken into account in addition to the morphology of tumors [1, 66, 71].

### 4.3. CLIP (Cancer of the Liver Italian Program) Classification

made from a retrospective analysis of 1990 to 1992 of parameters influencing survival of HCC patients and validated by authors and others groups in several prospective studies, CLIP score combines parameters predictive of survival: severity of liver disease (Child-Pugh score Tables 2 and 3), and tumor characteristics as tumor morphology, AFP rate, existence of portal vein thrombosis and classifies patients with HCC into six groups. Compared to the Okuda or TNM, Clip improves the prognostic accuracy because all predictor variables are considered as adverse prognostic characteristic in patients with HCC. CLIP has limitations when considered in HCC patients diagnosed in the early stage; survival difference for patient groups 4 to 6 is less discriminatory [68, 72–74]. 

### 4.4. BCLC (Barcelona Clinic Liver Center)

 This staging system classifies patients according to the evolutionary course of the tumor and liver disease, thus allowing for a confident predictive life expectation and choice of convenient treatment modality for patients in each group [75]. Very early/early stage (A) with asymptomatic early tumors (single tumor less than 5 cm), without portal hypertension, and without abnormal bilirubin are suitable for radical therapies such as resection, transplantations, or percutaneous treatments. Intermediate stage (B) with asymptomatic multinodular HCC is suitable for chemoembolization treatment. In advanced stage (C) with symptomatic tumors and/or an invasive tumoral pattern (alteration of the general state/portal vein thrombosis/metastases), the sorafenib is the standard treatment proposed. In end-stage disease with patients in a terminal stage of HCC, symptomatic care is most appropriate [76–78].

**Figure 2:**
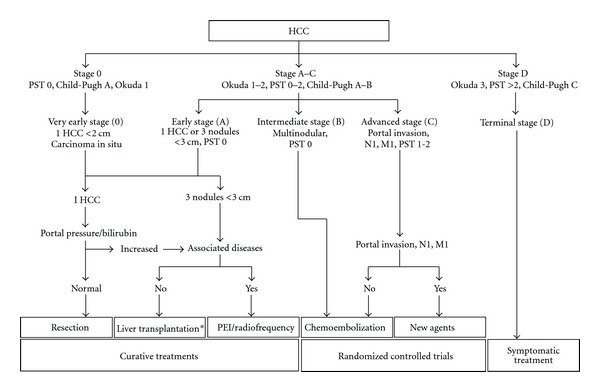
Barcelona-Clínic Liver Cancer staging classification and treatment schedule. PST: performance status test. N: nodules. M: metastases. PEI: percutaneous ethanol injection. *Cadaveric liver transplantation or living donor liver transplantation. (Source [78] with permission of The Lancet Journal.)

 BCLC is the only system of stratification in which each group is correlated with a mode of specific treatment (Figure 2). This advantage of linking the stage of HCC patient with appropriate treatment allows it to offer as the best classification system validated especially in patients with early HCC [68].

### 4.5. French Classification

Five factors prognostics are taken into account in classifying patient with HCC: Karnofsky index, serum bilirubin reflecting liver excretion or biliary function, serum alkaline phosphatase related to the growth rate of HCC, serum alpha-fetoprotein reflecting the degree of cellular differentiation and then the spreading of the tumor, and ultrasonographic portal obstruction. The prospective study was done in seven hundred and sixty-one (761) patients with hepatocellular carcinoma from 24 western medical centers enrolled over a 30-month period (from 15 July 1990 to 1 December 1992). Patients were divided into three groups (A, B, C) according to the increasing estimated risk of death (for instance, less than 5% at 2 years in group C and more than 50% in group A). This classification performed well as a single independent predictor of survival in the nonsurgical group according to Cox's regression analysis, probably because it also considers the patient's general health (Karnofsky index), unlike the Okuda, CLIP, and CUPI scores. Because of not taking into account tumor extension variables, it has limited prognostic capacity in patients with early HCC [68, 79].

### 4.6. CUPI (Chinese University Prognostic Index)

It has been investigated in Hong Kong from 1996 to 1998 on 926 ethnic Chinese patients who were diagnosed with HCC. Hepatitis B was detected in 79% of patients. In this system, patients were divided into three different groups, and six prognosis factors were taken into account by adding the following factors into the TNM staging system: total bilirubin, ascites, alkaline phosphatase, alfa-fetoprotein, and asymptomatic disease on presentation. As a conclusion, in patients with mainly hepatitis B-associated HCC, the CUPI was more discriminant than the TNM staging system, the Okuda staging systems, or the CLIP prognostic score in classifying patients into different risk groups and was better at predicting survival. However, for wide usage, the CUPI needs to be validated by different cohorts of patients [80].

### 4.7. JIS Score (Japan Integrated Staging)

It is a new staging system proposed in Japan and based on the Liver Cancer Study Group of Japan (LCSGJ). It has been investigated in a total of 722 patients with HCC covering a period of ten years. It accounts for both Child-Pugh classification and Japan tumour node metastasis (TNM) staging. The JIS scoring is a staging system developed to classify early-stage HCC patients requiring curative treatment, such as surgery and medical ablation. The prognostic predictive power of the JIS score was equal to the CLIP score and was better than the original BCLC staging classification [72, 81–83].

A consensus seems to be necessary to standardize the management of patients with hepatocellular carcinoma. For that, new strategies must be implemented in diversifying the classification criteria. Histological causes and molecular changes leading to HCC should provide new tools to be included in the stratification criteria.

## 5. HCC Diagnosis and Surveillance 

HCC diagnosis is considerable interest to clinician's evaluation patients with liver cirrhosis [84]. Generally, HCC appears with setting of cirrhosis with underlying chronic viral hepatitis (B or C) or alcoholism and more recently with nonalcoholic steatohepatitis [85]. In many patients, HCC is asymptomatic and then is diagnosed in an advanced stage. That is why, for cirrhotic patients, surveillance is strongly recommended to detect early HCC allowing an increase of patients suitable for curative treatment and then limit tumor-related dead. Symptoms of HCC are commonly related to those of their chronic liver disease and include pain in the upper abdomen on the right side, a lump or a feeling of heaviness in the upper abdomen, swollen abdomen (bloating), anemia, weight loss, weakness or fatigue, nausea and vomiting, yellowing skin and eyes, pale stools, and dark urine from jaundice caused by invasion of the biliary tree, fever, pain born in case of metastases [86–88]. 

The diagnosis criteria of HCC consist in the detection of the index lesion, intrahepatic lesion staging, and an assessment for extrahepatic metastasis [84]. An international consensus statement has been formulated by the EASL (European Association for the Study of Liver) to regulate the diagnosis approach and surveillance algorithm for HCC patients and has been updated in the American Association for the Study of Liver Disease 2005 guidelines (AASLD) [89, 90]. Identification of HCC in patients with cirrhosis was based on the radiological and histological criteria, and recommendations upon detection of nodular lesion during US surveillance were based on the size of the nodule [91]. 

AFP and live Us are widely used for HCC surveillance that must be done every 6 months since this time is considered to be the average time for tumor duplication [92]. AFP is used as a screening test because HCC may secrete elevated rate of AFP. Many studies of AFP in HCC surveillance revealed that its sensitivity is estimated at 39-64%, it specificity to 76–91%, and its positive predictive value to 9–32%. The specificity and sensitivity depend on the AFP cut-off level chosen for the diagnosis. A cut-off value of 20 ng/mL corresponded in a sensitivity of 64% and a specificity of 91%, while a sensitivity of 17% and specificity of 99% for the cut-off value of 400 ng/mL. Generally, diagnosis confirms HCC in values over 400 ng/mL, although this value was only found in 20% of HCC patients. AFP level is usually correlated to tumor morphology. Tumor less than 2 cm is rarely detectable. For these remarks, AFP is not considered as a suitable test [5, 18, 88, 93]. Us is much better screening tool than AFP. Since new methods are introduced such as contrast-enhanced Us, detection has been improved with better sensitivity and specificity [88, 94]. Cost-effectiveness of surveillance for early detection is debatable. It is found to allow patients diagnosis in a resectable stage improving then the long-term survival [89, 95]. 

EASL diagnosis approach proposed for nodules >2 cm noninvasive diagnosis of HCC can be done with arterial hypervascularization into two modalities, or in one imaging technique. Associated with an AFP level ≥400 ng/mL in cirrhotic liver, Us, spiral CT, MRI, and angiography are used to evaluate the vascularity of the hepatic nodules. Under these conditions, biopsy is not required avoiding the 10–20% false negative rate from histological samples and the risk for tumor seeding. 

 Nodules <1 cm, found to be malignant in less than 50% of cases and difficult to be effectively diagnosis even by biopsy, a repeated ultrasound screening every 3 months, are recommended until the 1 cm size is reached. 

Nodules between 1 and 2 cm are more likely to be HCC. Two imaging dynamic studies can be considered to be HCC, and diagnosis confirmation is required using biopsy or fine-needle aspiration or both of them. In these nodules 30-40% false negative rate are generally noted with a tumor seeding [4, 87–89, 91, 93, 96, 97]. 

## 6. Therapy

Therapeutic approaches are conventionally classified as curative and palliative. The choice of treatment depends on the tumor characteristics, liver function, and presence or not of metastasis or vascular invasion. Curative treatments are surgical resection, liver transplantation, and percutaneous ablation and aim to improve survival. Palliative approaches include systemic chemotherapy, immunotherapy, and hormonal compounds. Curative options can be considered in early diagnosis of HCC, which is generally defined as a HCC with a solitary tumor <5 cm, or up to 3 nodules <3 cm each with a good preserved hepatic function. However, just 1/3 of HCC patients will be candidates from curative treatment. In advanced stage, local extrahepatic spread, distant metastases, or in HCC patients not eligible for surgical approaches, palliative therapies are proposed. 

### 6.1. Liver Resection

Noncirrhotic patients or Child-Pugh A cirrhotic patients with a well-preserved liver function, a single nodule, and normal portal pressure are eligible for surgical resection [98–100]. As a result of these guidelines few HCC patients benefit from this method. In Asia where HCC has a high incidence, only 10–15% of newly diagnosed patients undergo resection therapy. In Western countries, only 5–10% are candidates for resection surgery. Differences in tumor biology, in health-care standards, and the HCC-related etiology which is HBV-infection especially in Asia explain in large part these comments [13, 41]. Long-term survival of 5 years can be achieved in 60–70% of patients who have undergone resection depending on the stage of disease. After treatment with surgical intent, there is a very high risk of recurrence, 70% at 5 years, and within 2 years in majority. Recurrence reasons are complex and depend on the size and differentiation of tumor, intrahepatic metastases undetectable at resection time, vascular invasion, and satellites nodules. A repeated resection is generally possible in only 20% of cases. Therefore, these patients should be evaluated for effective prevention of recurrence using adjuvant treatment such as interferon, lipiodol, and adoptive immunotherapy, retinoids (polyprenoic acid) even if these promising results require further validation [20, 88, 89, 93, 101, 102]. 

### 6.2. Liver Transplantation

Orthotopic liver transplantation (OLT) is the curative options in which both the tumor and the underlying liver disease are removed. Therefore, it is the treatment of choice for patients with hepatic cirrhosis-related hepatocellular carcinoma [103–105]. Patients with HCC meeting the Milan criteria [106] which are single nodule ≤5 cm in diameter or up to 3 separate lesions all less than 3 cm, no proven vascular invasion, no nodal or distant metastases are appropriated candidates for OLT. Under these criteria, an overall and recurrence-free survival rates at 4 years of 85% and 92% have been shown. With these convincing results, much research has been done to improve the criteria for selecting patients for OLT. Using less stringent criteria, which are a maximum nodule size of 6.5 cm or 2 lesions <4.5 cm diameter with a total tumor diameter <8 cm, the group from the University of California at San Francisco (UCSF) has demonstrated a 5-year survival of 75% [107]. In most of the liver transplantation, the liver is from a deceased donor. This constitutes a major drawback of this treatment because of the scarcity of donors that is crucial in Asia. As a consequence, the MELD (Model of End-Stage liver Disease) scoring system has been adopted to allocate organs for the patient in waiting list for liver transplantation. This score can range from 0 to >40 points and is indicative of the risk of death without liver transplantation. Patients with higher score are priority and receive the first offers [108–112]. To overcome the scarcity of deceased donors, the notion of living donor liver transplantation (LDLT) has been adopted and has allowed to face long waiting times for deceased donor liver grafts. Several retrospective studies have shown that LDLT for HCC has a similar survival to that of deceased donor transplantation [113, 114].

### 6.3. Percutaneous Ablation

Patients with early HCC who are not candidates for resection or liver transplantation, are treated preferentially with percutaneous ablation, that is considered as a minimally invasive option with low mortality, low rates of complications, and good outcomes in overall survival [115–117]. Percutaneous ethanol injection (PEI) and radiofrequency thermal ablation (RFA) are the most widely procedures used in ablation therapy and are done under guidance imaging. PEI was the first standard ablation treatment, but few years later RFA was recommended as the best option for percutaneous ablation. In most institutions in Asia, RFA has replaced the PEI [13, 89, 118]. New ablative therapies have been introduced such as microwave ablation, cryoablation, and high-intensity focused ultrasound ablation. Child-Pugh A patients are the best candidates with a 5-year survival of 50%. The most frequent drawback for percutaneous is the high rate of recurrence which is evaluated in PEI at 33–43% [117]. 

### 6.4. Palliative Therapy

Due to the implementation of prevention programs for HCC, patients are usually diagnosed in early stage. Nevertheless, some are detectable at an intermediate or advanced stage with preserved liver function or not, multinodular disease exceeding the Milan criteria, with or without symptoms or extrahepatic spread manifested by absence of portal/node invasion or distant metastasis. For these patients, palliative therapies are proposed to reduce symptoms related to disease progression and improve survival [89, 118]. The general principle of palliative treatment is to prevent the blood supply to the HCC by blockage of the arterial system of liver. These treatments include transarterial embolization (TAE), transarterial chemoembolization (TACE), intra-artery chemotherapy (IACT), and radiotherapy (external and internal) [119, 120]. They can be used as bridge to liver transplantation. 

 TAE and IACT are known to have an anticancer activity but no effect in increasing survival [4]. TACE is the most widely used for unrespectable HCC and one of the most used techniques to control liver cancer around the world. [121]. Intra-arterial delivery of chemotherapeutic agents (especially doxorubicin combined with others agents) and blocking (embolizing) the small blood vessels feeding the tumor are the process treatment used by TACE. Patients with multiple diffuse tumors or uninodular larger than 5 cm, well preserved liver function, and absence of portal vein invasion or Child-Turcott Plug class C are main candidates for this treatment [87]. Patient's selection is essential for TACE because it avoid side effects leading to liver failure and death [89]. Randomized control trials have shown benefit of TACE. An objective response lasting 1–6 months in 35% of patients and improvement survival rate in unresectable HCC have been proven [4]. 

Patients in intermediate stage to advanced stage with portal vein thrombosis (PVT) can be treated by yttrium-90 radioembolization (90Y-RE). It consists of direct injection into the blood vessels feeding the cancers with a radioactive molecule yttrium-90 impregnated glass microspheres or resin beads. The radiation particles can then kill tumor cells within a distance of 2.5 mm from them so that any part of the cancer fed by tiny blood vessels will be exposed to the radiation. Hence, tumor growth is inhibited and liver function remains preserved. Recipient patients respond to treatment with an improved quality of life. It can be used as bridge to RFA and resection. Comparison between the survival of HCC patients in advanced stage either not treated or treated with ineffective systemic agents, survival after (90)Y-RE is encouraging and warrants future clinical trials [122–125]. 

### 6.5. Systemic Therapies

Parallel to the development of research concerning pathological and histological pathways leading to HCC, many drugs and hormonal therapy have been tested as agents inhibiting important signaling pathways in tumor cells and also angiogenesis in systemic therapy for HCC. Patients in intermediate or advanced stage who do not undergo curative surgical treatment and ablative techniques are candidates for these therapies. Systemic therapies have not unfortunately provided benefit effect or increased survival for patients with advanced HCC. Several clinical trials have been conducted on these agents searching an optimal therapy against advanced HCC, but there is no currently defined as a standard formula effective against advanced HCC. They can be used as single or combined hormonal or chemotherapy. The most used is the multikinase inhibitor sorafenib that was approved by the European Medicines Agency (EMEA) and the US Food and Drug Administration (FDA). These promising benefits in patients with metastatic disease need further evaluation. Other therapeutic agents such as doxorubicin, epirubicin, mitoxantrone, cisplatin, gemcitabine, capecitabine, 5-Flu, Tamoxifen, and placebo. have been also used as single agent against advanced HCC. Some combined agents against HCC have been investigated, and the most known are cisplatin, doxorubicin, 5-Flu, and interferon-*α* (PIAF), gemcitabine and oxaliplatin (GEMOX), oxaliplatin and 5-Flu/leucovorin (FOLFOX), capecitabine and oxaliplatin (XELOX). The results vary depending on the research groups, countries, and characteristics of the patients tested [10, 41, 126–130].

## 7. Conclusion

The HCC remains a malignant disease leading to death. Significant progress has been made in the management of the disease. Because of its complexity, a multidisciplinary approach must be implemented to support the different aspects in HCC. A better control of epidemiology should better sit prevention programs in at-risk populations. A better understanding of the molecular and histological responsible for the occurrence of the disease should allow the development of new diagnostics and treatments more effective in the treatment of HCC. 

## Figures and Tables

**Table 1:** Staging system and prognostic variables used in the staging systems in HCC (adapted from [68] with permission of the HPB (Oxford) journal).

Classification	Type	Stage	Tumor stage	Liver function	Health status
Okuda stage	System	Stage I, II, III	50% liver involvement	Bilirubin Albumin Ascites	—
French	Score	A: 0 points; B: 1–5 points; C: 56 points	Portal invasion AFP	Bilirubin Alkaline phosphatase	Karnofsky
CLIP	Score	0, 1, 2, 3, 4, 5, 6	Portal invasion 5/450% liver involvement AFP	Child-Pugh	—
BCLC staging	Staging	0: Very early A: Early B: Intermediate C: Advanced D: End-stage	Portal invasion Metastases Morphology Okuda	Child-Pugh Portal hypertension Bilirubin	PST
TNM staging	System	Stage I, II, III	Morphology Vascular invasion Metastases	Fibrosis	—
CUPI	Score	Low risk: score ≤1 Intermediate: score 2–7 High: score ≥ 8	TNM AFP	Ascites Bilirubin	Symptoms
JIS	Score	Stage I, II, III, IV	TNM	Child-Pugh	—

**Table 2:** Variables used in the Child-Pugh score (source [8]).

Measure	1 point	2 points	3 points
Total bilirubin, *μ*moL/L (mg/dL)	<34 (<2)	34–50 (2-3)	>50 (>3)
Serum albumin, g/L	>35	28–35	<28
INR	<1.7	1.71–2.20	>2.20
Ascites	None	Mild	Severe
Hepatic encephalopathy	None	Grade I-II (or suppressed with medication)	Grade III-IV (or refractory)

**Table 3:** Interpretation of the Child-Pugh score (source [8]).

Points	Class	One-year survival	Two-year survival
5-6	A	100%	85%
7–9	B	81%	57%
10–15	C	45%	35%

## References

[1] Yang JD, Roberts LR (2010). Epidemiology and management of hepatocellular carcinoma. *Infectious Disease Clinics of North America*.

[2] Gomaa AI, Khan SA, Toledano MB, Waked I, Taylor-Robinson SD (2008). Hepatocellular carcinoma: epidemiology, risk factors and pathogenesis. *World Journal of Gastroenterology*.

[3] McGlynn KA, London WT (2005). Epidemiology and natural history of hepatocellular carcinoma. *Best Practice and Research*.

[4] Mazzanti R, Gramantieri L, Bolondi L (2008). Hepatocellular carcinoma: epidemiology and clinical aspects. *Molecular Aspects of Medicine*.

[5] Shariff MIF, Cox IJ, Gomaa AI, Khan SA, Gedroyc W, Taylor-Robinson SD (2009). Hepatocellular carcinoma: current trends in worldwide epidemiology, risk factors, diagnosis and therapeutics. *Expert Review of Gastroenterology and Hepatology*.

[6] Kew MC (2010). Epidemiology of chronic hepatitis B virus infection, hepatocellular carcinoma, and hepatitis B virus-induced hepatocellular carcinoma. *Pathologie Biologie*.

[7] Schütte K, Bornschein J, Malfertheiner P (2009). Hepatocellular carcinoma-epidemiological trends and risk factors. *Digestive Diseases*.

[8] Child-Pugh Score http://en.wikipedia.org/wiki/Child-Pugh_score.

[9] Hadziyannis SJ (2011). Natural history of chronic hepatitis B in Euro-Mediterranean and African countries. *Journal of Hepatology*.

[10] Hainaut P, Boyle P (2008). Curbing the liver cancer epidemic in Africa. *The Lancet*.

[11] Liu J, Fan D (2007). Hepatitis B in China. *The Lancet*.

[12] Ferenci P, Fried M, Labrecque D (2010). W2010orld gastroenterology organization guideline. Hepatocellular carcinoma (HCC): a global perspective. *Journal of Gastrointestinal and Liver Diseases*.

[13] Poon D, Anderson BO, Chen LT (2009). Management of hepatocellular carcinoma in Asia: consensus statement from the Asian Oncology Summit 2009. *The Lancet Oncology*.

[14] Yuen MF, Hou JL, Chutaputti A (2009). Hepatocellular carcinoma in the Asia pacific region. *Journal of Gastroenterology and Hepatology*.

[15] Umemura T, Ichijo T, Yoshizawa K, Tanaka E, Kiyosawa K (2009). Epidemiology of hepatocellular carcinoma in Japan. *Journal of Gastroenterology*.

[16] Sherman M (2010). Epidemiology of hepatocellular carcinoma. *Oncology*.

[17] Okuda K (2000). Hepatocellular carcinoma. *Journal of Hepatology*.

[18] Ahn J, Flamm SL (2004). Hepatocellular Carcinoma. *Disease-a-Month*.

[19] Nordenstedt H, White DL, El-Serag HB (2010). The changing pattern of epidemiology in hepatocellular carcinoma. *Digestive and Liver Disease*.

[20] Bruix J, Boix L, Sala M, Llovet JM (2004). Focus on hepatocellular carcinoma. *Cancer Cell*.

[21] Okuda H (2007). Hepatocellular carcinoma development in cirrhosis. *Best Practice and Research*.

[22] Coon JT, Rogers G, Hewson P (2007). Surveillance of cirrhosis for hepatocellular carcinoma: systematic review and economic analysis. *Health Technology Assessment*.

[23] Rosen HR (2011). Clinical practice. Chronic hepatitis C infection. *The New England Journal of Medicine*.

[24] Chiesa R, Donato F, Tagger A (2000). Etiology of hepatocellular carcinoma in Italian patients with and without cirrhosis. *Cancer Epidemiology Biomarkers and Prevention*.

[25] Bartolomeo N, Trerotoli P, Serio G (2011). Progression of liver cirrhosis to HCC: an application of hidden Markov model. *BMC Medical Research Methodology*.

[26] Tabor E (2001). Hepatocellular carcinoma: global epidemiology. *Digestive and Liver Disease*.

[27] Nalpas B, Pol S, Thépot V, Berthelot P, Brechot C (1995). Hepatocellular carcinoma in alcoholics. *Alcohol*.

[28] Fartoux L, Desbois-Mouthon C, Rosmorduc O Carcinome hépatocellulaire: épidémiologie, physiopathologie et diagnostic.

[29] Hassan MM, Kaseb AO, McMasters KM, Vauthey JN (2011). Epidemiology and pathogenesis of hepatocellular carcinoma. *Hepatocellular Carcinoma*.

[30] Morgan TR, Mandayam S, Jamal MM (2004). Alcohol and hepatocellular carcinoma. *Gastroenterology*.

[31] Liu Y, Wu F (2010). Global burden of Aflatoxin-induced hepatocellular carcinoma: a risk assessment. *Environmental Health Perspectives*.

[32] Smela ME, Currier SS, Bailey EA, Essigmann JM (2001). The chemistry and biology of aflatoxin B1: from mutational spectrometry to carcinogenesis. *Carcinogenesis*.

[33] Lasky T, Magder L (1997). Hepatocellular carcinoma p53 G > T transversions at codon 249: the fingerprint of aflatoxin exposure?. *Environmental Health Perspectives*.

[34] Wang JS, Huang T, Su J (2001). Hepatocellular carcinoma and aflatoxin exposure in Zhuqing Village, Fusui County, People’s Republic of China. *Cancer Epidemiology Biomarkers and Prevention*.

[35] Mori S, Yamasaki T, Sakaida I (2004). Hepatocellular carcinoma with nonalcoholic steatohepatitis. *Journal of Gastroenterology*.

[36] Watanabe S, Horie Y, Kataoka E (2007). Non-alcoholic steatohepatitis and hepatocellular carcinoma: lessons from hepatocyte-specific phosphatase and tensin homolog (PTEN)-deficient mice. *Journal of Gastroenterology and Hepatology*.

[37] Takuma Y, Nouso K (2010). Nonalcoholic steatohepatitis-associated hepatocellular carcinoma: our case series and literature review. *World Journal of Gastroenterology*.

[38] Chagas AL, Kikuchi LOO, Oliveira CPMS (2009). Does hepatocellular carcinoma in non-alcoholic steatohepatitis exist in cirrhotic and non-cirrhotic patients?. *Brazilian Journal of Medical and Biological Research*.

[39] Bugianesi E (2007). Non-alcoholic steatohepatitis and cancer. *Clinics in Liver Disease*.

[40] Masuzaki R, Yoshida H, Tateishi R, Shiina S, Omata M (2008). Hepatocellular carcinoma in viral hepatitis: improving standard therapy. *Best Practice and Research*.

[41] Wörns MA, Galle PR (2010). Future perspectives in hepatocellular carcinoma. *Digestive and Liver Disease*.

[42] Roncalli M, Park YN, di Tommaso L (2010). Histopathological classification of hepatocellular carcinoma. *Digestive and Liver Disease*.

[43] Chang M-H http://www.cchi.com.hk/symposia/s4_hepatitis.htm#2.

[44] Chang MH, Chen CJ, Lai MS (1997). Universal hepatitis B vaccination in Taiwan and the incidence of hepatocellular carcinoma in children. *New England Journal of Medicine*.

[45] Poovorawan Y, Theamboonlers A, Vimolket T (2000). Impact of hepatitis B immunization as part of the EPI. *Vaccine*.

[46] Lim SG, Mohammed R, Yuen MF, Kao JH (2009). Prevention of hepatocellular carcinoma in hepatitis B virus infection. *Journal of Gastroenterology and Hepatology*.

[47] Schott E, Bergk A, Berg T (2008). Strategies for the prevention of hepatocellular carcinoma in the context of chronic viral hepatitis. *Zeitschrift fur Gastroenterologie*.

[48] Kew MC (2011). Prevention of hepatocellular carcinoma. *South African Journal of Surgery*.

[49] Liu CJ, Kao JH (2007). Hepatitis B virus-related hepatocellular carcinoma: epidemiology and pathogenic role of viral factors. *Journal of the Chinese Medical Association*.

[50] Zucman-Rossi J (2010). Molecular classification of hepatocellular carcinoma. *Digestive and Liver Disease*.

[51] Lee HC, Kim M, Wands JR (2006). Wnt/frizzled signaling in hepatocellular carcinoma. *Frontiers in Bioscience*.

[52] Cavard C, Colnot S, Audard V (2008). Wnt/*β*-catenin pathway in hepatocellular carcinoma pathogenesis and liver physiology. *Future Oncology*.

[53] Takigawa Y, Brown AM (2008). Wnt signaling in liver cancer. *Current drug targets*.

[54] Zhang XF, Yu L, Lu Y (2008). Wnt/*β*-catenin signaling pathway and its role in hepatocellular carcinoma. *Frontiers of Medicine in China*.

[55] Samani AA, Yakar S, LeRoith D, Brodt P (2007). The role of the IGF system in cancer growth and metastasis: overview and recent insights. *Endocrine Reviews*.

[56] Lachenmayer A, Alsinet C, Chang CY, Llovet JM (2010). Molecular approaches to treatment of hepatocellular carcinoma. *Digestive and Liver Disease*.

[57] Desbois-Mouthon C, Baron A, Blivet-Van Eggelpoël MJ (2009). Insulin-like growth factor-1 receptor inhibition induces a resistance mechanism via the epidermal growth factor receptor/HER3/AKT signaling pathway: rational basis for cotargeting insulin-like growth factor-1 receptor and epidermal growth factor receptor in hepatocellular carcinoma. *Clinical Cancer Research*.

[58] Chen JS, Wang Q, Fu XH (2009). Involvement of PI3K/PTEN/AKT/mTOR pathway in invasion and metastasis in hepatocellular carcinoma: association with MMP-9. *Hepatology Research*.

[59] Paez J, Sellers WR (2003). PI3K/PTEN/AKT pathway. A critical mediator of oncogenic signaling.. *Cancer treatment and research*.

[60] Villanueva A, Hoshida Y (2011). Depicting the role of TP53 in hepatocellular carcinoma progression. *Journal of Hepatology*.

[61] Puisieux A, Ozturk M (1997). TP53 and hepatocellular carcinoma. *Pathologie Biologie*.

[62] Guan YS, He O, La Z (2006). Roles of p53 in carcinogenesis, diagnosis and treatment of hepatocellular carcinoma. *Journal of Cancer Molecules*.

[63] Hussain SP, Schwank J, Staib F, Wang XW, Harris CC (2007). TP53 mutations and hepatocellular carcinoma: insights into the etiology and pathogenesis of liver cancer. *Oncogene*.

[64] Besaratinia A, Kim SI, Hainaut P, Pfeifer GP (2009). In vitro recapitulating of TP53 mutagenesis in hepatocellular carcinoma associated with dietary aflatoxin B_1_ exposure. *Gastroenterology*.

[65] Gouas DA, Shi H, Hautefeuille AH (2010). Effects of the TP53 p.R249S mutant on proliferation and clonogenic properties in human hepatocellular carcinoma cell lines: Interaction with hepatitis B virus X protein. *Carcinogenesis*.

[66] Yan P, Yan LN (2003). Staging of hepatocellular carcinoma. *Hepatobiliary and Pancreatic Diseases International*.

[67] Marrero JA, Fontana RJ, Barrat A (2005). Prognosis of hepatocellular carcinoma: comparison of 7 staging systems in an American cohort. *Hepatology*.

[68] Pons F, Varela M, Llovet JM (2005). Staging systems in hepatocellular carcinoma. *Hepato-Pancreato-Biliary Journal*.

[69] Martin AP (2009). Management of hepatocellular carcinoma in the age of liver transplantation. *International Journal of Surgery*.

[70] Dilou N, Patouillard B, Audigier JC (2004). Staging systems in hepatocellular carcinoma. *Gastroenterologie Clinique et Biologique*.

[71] Hermanek P, Sobin LH, Wittekind C (1999). How to improve the present TNM staging system. *Cancer*.

[72] Kudo M, Chung H, Osaki Y (2003). Prognostic staging system for hepatocellular carcinoma (CLIP score): its value and limitations, and a proposal for a new staging system, the Japan integrated staging score (JIS score). *Journal of Gastroenterology*.

[73] Zhao WH, Ma ZM, Zhou XR, Feng YZ, Fang BS (2002). Prediction of recurrence and prognosis in patients with hepatocellular carcinoma after resection by use of CLIP score. *World Journal of Gastroenterology*.

[74] Cammà C, di Marco V, Cabibbo G (2008). Survival of patients with hepatocellular carcinoma in cirrhosis: a comparison of BCLC, CLIP and GRETCH staging systems. *Alimentary Pharmacology and Therapeutics*.

[75] Iavarone M, Colombo M (2011). HBV-related HCC, clinical issues and therapy. *Digestive and Liver Disease*.

[76] Llovet JM, Brú C, Bruix J (1999). Prognosis of hepatocellular carcinoma: the BCLC staging classification. *Seminars in Liver Disease*.

[77] Forner A, Reig ME, de Lope CR, Bruix J (2010). Current strategy for staging and treatment: the BCLC update and future prospects. *Seminars in Liver Disease*.

[78] Llovet JM, Burroughs A, Bruix J (2003). Hepatocellular carcinoma. *The Lancet*.

[79] Chevret S, Trinchet JC, Mathieu D, Rached AA, Beaugrand M, Chastang C (1999). A new prognostic classification for predicting survival in patients with hepatocellular carcinoma. *Journal of Hepatology*.

[80] Leung TWT, Tang AMY, Zee B (2002). Construction of the Chinese University Prognostic Index for hepatocellular carcinoma and comparison with the TNM staging system, the Okuda staging system, and the Cancer of the Liver Italian Program staging system: a study based on 926 patients. *Cancer*.

[81] Nanashima A, Sumida Y, Morino S (2004). The Japanese integrated staging score using liver damage grade for hepatocellular carcinoma in patients after hepatectomy. *European Journal of Surgical Oncology*.

[82] Cillo U, Bassanello M, Vitale A (2004). The critical issue of hepatocellular carcinoma prognostic classification: which is the best tool available?. *Journal of Hepatology*.

[83] Chung H, Kudo M, Takahashi S (2008). Comparison of three current staging systems for hepatocellular carcinoma: Japan integrated staging score, new Barcelona Clinic Liver Cancer staging classification, and Tokyo score. *Journal of Gastroenterology and Hepatology*.

[84] Talwalkar JA, Gores GJ (2004). Diagnosis and staging of hepatocellular carcinoma. *Gastroenterology*.

[85] Saar B, Kellner-Weldon F (2008). Radiological diagnosis of hepatocellular carcinoma. *Liver International*.

[86] Sala M, Bruix J (2004). *Management of Hepatocellular Carcinoma. Management of Patients with Viral Hepatitis*.

[87] Naugler WE, Schwartz JM (2008). Hepatocellular carcinoma. *Disease-a-Month*.

[88] El-Serag HB, Marrero JA, Rudolph L, Reddy KR (2008). Diagnosis and treatment of hepatocellular carcinoma. *Gastroenterology*.

[89] Bruix J, Sherman M, Llovet JM (2001). Clinical management of hepatocellular carcinoma. Conclusions of the Barcelona-2000 EASL conference. European Association for the Study of the Liver. *Journal of Hepatology*.

[90] Bruix J, Sherman M (2005). Management of hepatocellular carcinoma. *Hepatology*.

[91] Gomaa AI, Khan SA, Leen ELS, Waked I, Taylor-Robinson SD (2009). Diagnosis of hepatocellular carcinoma. *World Journal of Gastroenterology*.

[92] França AVC, Junior JE, Lima BLG, Martinelli ALC, Carrilho FJ (2004). Diagnosis, staging and treatment of hepatocellular carcinoma. *Brazilian Journal of Medical and Biological Research*.

[93] Bruix J, Llovet JM (2003). Hepatitis B virus and hepatocellular carcinoma. *Journal of Hepatology*.

[94] Lencioni R, Della Pina C, Cioni D, Crocetti L (2008). Guidelines for the use of contrast-enhanced ultrasound in hepatocellular carcinoma. *European Journal of Cancer Supplements*.

[95] Cha CH, Saif MW, Yamane BH, Weber SM (2010). Hepatocellular carcinoma: current management. *Current Problems in Surgery*.

[96] Ryder SD (2003). Guidelines for the diagnosis and treatment of hepatocellular carcinoma (HCC) in adults. *Gut*.

[97] Lencioni R (2010). Surveillance and early diagnosis of hepatocellular carcinoma. *Digestive and Liver Disease*.

[98] Capussotti L, Ferrero A, Viganò L, Polastri R, Tabone M (2009). Liver resection for HCC with cirrhosis: surgical perspectives out of EASL/AASLD guidelines. *European Journal of Surgical Oncology*.

[99] Delis S, Bakoyiannis A, Papailiou J (2009). Liver resection vs radio-frequency ablation in the treatment of small hepatocellular carcinoma. *Surgical Oncology*.

[100] Andreana L, Isgrò G, Pleguezuelo M (2009). Surveillance and diagnosis of hepatocellular carcinoma in patients with cirrhosis. *World Journal of Hepatology*.

[101] Duffy JP, Hiatt JR, Busuttil RW (2008). Surgical resection of hepatocellular carcinoma. *Cancer Journal*.

[102] Qin L, Tang Z (2005). Metastasis and recurrence after surgical resection of hepatocellular carcinoma: recent progress in clinical and related basic aspects. *Current Cancer Therapy Reviews*.

[103] González-Uriarte J, Valdivieso A, Gastaca M (2003). Liver transplantation for hepatocellular carcinoma in cirrhotic patients. *Transplantation Proceedings*.

[104] Tanwar S, Khan SA, Grover VPB, Gwilt C, Smith B, Brown A (2009). Liver transplantation for hepatocellular carcinoma. *World Journal of Gastroenterology*.

[105] Mazzaferro V, Regalia E, Doci R (1996). Liver transplantation for the treatment of small hepatocellular carcinomas in patients with cirrhosis. *New England Journal of Medicine*.

[106] Mazzaferro V, Chun YS, Poon RTP (2008). Liver transplantation for hepatocellular carcinoma. *Annals of Surgical Oncology*.

[107] Yao FY, Xiao L, Bass NM, Kerlan R, Ascher NL, Roberts JP (2007). Liver transplantation for hepatocellular carcinoma: validation of the UCSF-expanded criteria based on preoperative imaging. *American Journal of Transplantation*.

[108] Mak KSW, Tan KC (2002). Liver transplantation for hepatocellular carcinoma: an Asian perspective. *Asian Journal of Surgery*.

[109] Suehiro T, Terashi T, Shiotani S, Soejima Y, Sugimachi K (2002). Liver transplantation for hepatocellular carcinoma. *Surgery*.

[110] Llovet JM, Bruix J (2008). Novel advancements in the management of hepatocellular carcinoma in 2008. *Journal of Hepatology*.

[111] Sharma P, Harper AM, Hernandez JL (2006). Reduced priority MELD score for hepatocellular carcinoma does not adversely impact candidate survival awaiting liver transplantation. *American Journal of Transplantation*.

[112] UNOS http://www.unos.org.

[113] Lo CM, Fan ST, Liu CL, Chan SC, Wong J (2004). The role and limitation of living donor liver transplantation for hepatocellular carcinoma. *Liver Transplantation*.

[114] Hwang S, Lee SG, Joh JW, Suh KS, Kim DG (2005). Liver transplantation for adult patients with hepatocellular carcinoma in Korea: comparison between cadaveric donor and living donor liver transplantations. *Liver Transplantation*.

[115] McWilliams JP, Yamamoto S, Raman SS (2010). Percutaneous ablation of hepatocellular carcinoma: current status. *Journal of Vascular and Interventional Radiology*.

[116] Francica G, Pacella CM (2007). Percutaneous laser ablation of small hepatocellular carcinoma. *Current Medical Imaging Reviews*.

[117] Lencioni R, Cioni D, Crocetti L (2004). Percutaneous ablation of hepatocellular carcinoma: state-of-the-art. *Liver Transplantation*.

[118] Bruix J, Llovet JM (1999). Locoregional treatments for hepatocellular carcinoma. *Best Practice and Research Clinical Gastroenterology*.

[119] Villanueva A, Newell P, Hoshida Y (2010). Inherited hepatocellular carcinoma. *Best Practice and Research Clinical Gastroenterology*.

[120] Liapi E, Geschwind JFH (2010). Intra-arterial therapies for hepatocellular carcinoma: where do we stand?. *Annals of Surgical Oncology*.

[121] Liver Cancer http://www.medicinenet.com/liver_cancer/page10.htm.

[122] Sangro B, Bilbao JI, Iñarrairaegui M, Rodriguez M, Garrastachu P, Martinez-Cuesta A (2009). Treatment of hepatocellular carcinoma by radioembolization using y microspheres. *Digestive Diseases*.

[123] Cao CQ, Yan TD, Bester L, Liauw W, Morris DL (2010). Radioembolization with yttrium microspheres for neuroendocrine tumour liver metastases. *British Journal of Surgery*.

[124] Carr BI, Kondragunta V, Buch SC, Branch RA (2010). Therapeutic equivalence in survival for hepatic arterial chemoembolization and yttrium 90 microsphere treatments in unresectable hepatocellular carcinoma: a two-cohort study. *Cancer*.

[125] Sangro B, Carpanese L, Cianni R (2011). Survival after Yttrium-90 resin microsphere radioembolization of hepatocellular carcinoma across barcelona clinic liver cancer stages: a European evaluation. *Hepatology*.

[126] Thomas MB, O’Beirne JP, Furuse J, Chan ATC, Abou-Alfa G, Johnson P (2008). Systemic therapy for hepatocellular carcinoma: cytotoxic chemotherapy, targeted therapy and immunotherapy. *Annals of Surgical Oncology*.

[127] Witjes CDM, Verhoef C, Verheul HMW, Eskens FALM (2009). Systemic treatment in hepatocellular carcinoma; ‘a small step for man...’. *Netherlands Journal of Medicine*.

[128] Zhu AX (2006). Systemic therapy of advanced hepatocellular carcinoma: how hopeful should we be?. *Oncologist*.

[129] Bruix J, Sherman M (2011). Management of hepatocellular carcinoma: an update. *Hepatology*.

[130] Sherman M (2011). Hepatocellular carcinoma: screening and staging. *Clinics in Liver Disease*.

